# The structure of post-traumatic stress disorder and complex post-traumatic stress disorder amongst West Papuan refugees

**DOI:** 10.1186/s12888-015-0480-3

**Published:** 2015-05-07

**Authors:** Alvin Kuowei Tay, Susan Rees, Jack Chen, Moses Kareth, Derrick Silove

**Affiliations:** 1 Psychiatry Research and Teaching Unit, Liverpool Hospital, School of Psychiatry, University of New South Wales, Sydney, Australia; 2 The Ingham Institute, Liverpool Hospital, Faculty of Medicine, University of New South Wales, Sydney, Australia; 3 Simpson Centre for Health Services Research, Faculty of Medicine, University of New South Wales, Sydney, Australia; 4 South Western Sydney Clinical School, Liverpool Hospital, Faculty of Medicine, University of New South Wales, Sydney, Australia

**Keywords:** Refugee, PTSD, Trauma, Nosology, Transcultural psychiatry

## Abstract

**Background:**

The validity of applying the construct of post-traumatic stress disorder (PTSD) across cultures has been the subject of contention. Although PTSD symptoms have been identified across multiple cultures, questions remain whether the constellation represents a coherent construct with an interpretable factor structure across diverse populations, especially those naïve to western notions of mental disorder. An important additional question is whether a constellation of Complex-PTSD (C-PTSD) can be identified and if so, whether there are distinctions between that disorder and core PTSD in patterns of antecedent traumatic events. Our study amongst West Papuan refugees in Papua New Guinea (PNG) aimed to examine the factorial structure of PTSD based on the DSM-IV, DSM-5, ICD-10 and ICD-11 definitions, and C-PTSD according to proposed ICD-11 criteria. We also investigated domains of traumatic events (TEs) and broader psychosocial effects of conflict (sense of safety and injustice) associated with the factorial structures identified.

**Methods:**

Culturally adapted measures were applied to assess exposure to conflict-related traumatic events (TEs), refugees’ sense of safety and justice, and symptoms of PTSD and C-PTSD amongst 230 West Papuan refugees residing in Port Morseby, PNG.

**Results:**

Confirmatory factor analysis (CFA) supported a unitary construct of both ICD-10 and ICD-11 PTSD, comprising the conventional symptom subdomains of intrusion, avoidance, and hyperarousal. In contrast, CFA did not identify a unitary construct underlying C-PTSD. The interaction of witnessing murders and sense of injustice was associated with both the intrusion and avoidance domains of PTSD, but not with the unique symptom clusters characterizing C-PTSD.

**Conclusions:**

Our findings support the ICD PTSD construct and its three-factor structure in this transcultural refugee population. Traumatic experiences of witnessing murder associated with a sense of injustice were specifically related to the intrusion and avoidance domains of PTSD. The unitary nature of C-PTSD across cultures remains in question.

**Electronic supplementary material:**

The online version of this article (doi:10.1186/s12888-015-0480-3) contains supplementary material, which is available to authorized users.

## Background

The psychiatric diagnosis of posttraumatic stress disorder (PTSD) has attracted ongoing controversy since its introduction in DSM-III [1,2]. Concerns have been raised about the tendency for the PTSD category to be applied to persons exposed to an ever-widening range of common-day life experiences such as exposure to severe illness, accidents and severe work stresses [3]. In contrast, early formulations of PTSD restricted the diagnosis to survivors of extreme events such as torture, political persecution and sexual assault [4].

Commentators also have raised questions about the validity of applying PTSD as a psychopathological entity across cultures [5]. At the extreme, critics have claimed that PTSD is culture-bound disorder to western societies, representing a social construct promoted for advocacy reasons, particularly during the Vietnam war, and further propagated by media publicity, mental health professionals, and the compensation industry [5-7]. The contrary perspective, supported by extensive research in the field, is that symptoms of PTSD can be identified across a wide range of cultures and contexts [8]. Nevertheless, demonstrating the presence of symptoms does not, *ipso facto*, offer definitive evidence in support of a psychopathological category. There is a pressing need therefore, to examine whether a common factorial structure can be identified underlying the PTSD construct when tested across culturally diverse populations, particularly amongst communities exposed to conflict and persecution who have had no or minimal exposure to western concepts of mental disorder or services providing treatment for traumatic stress.

Repeated changes to the diagnostic criteria of PTSD added to the complexity of identifying a common factorial structure underlying the disorder. In the Diagnostic and Statistical Manual Edition IV (DSM-IV), PTSD is defined according to the three symptom domains of re-experiencing, avoidance/numbing, and arousal [9]. In DSM-5, the avoidance and numbing constellations have been separated, generating a fourth domain of “persistent alterations in mood and cognition” [10]. In addition, studies examining the DSM-5 symptom constellation amongst war veterans and general community populations have supported a 5-factor dysphoric-arousal model, a 6-factor model identifying an irritability and self-destructive behaviour (“externalizing behaviour”) factor, and a 7-factor model that separates the domains of anhedonia and externalizing behavior [11-13]. In contrast, both ICD-10 and the proposed ICD-11 criteria limit the definition of PTSD to three core symptom clusters, namely re-experiencing (or intrusions), avoidance, and hyper-arousal, the latter system including a reduced number of symptoms for each domain [14].

A further area of complexity relates to the longstanding proposition that survivors of extreme traumas such as childhood sexual abuse, rape and torture are prone to experience a complex form of PTSD [15]. Implicit in the formulation of complex PTSD (C-PTSD) is that traumas such as rape and torture result in reactions such as anger, mistrust and interpersonal difficulties because of the humiliation, shame and degradation (encapsulated by a persisting sense of injustice), that these forms of intentional interpersonal abuse engender [15]. Nevertheless, there has been a reluctance to include a category of C-PTSD in international classification systems, largely because of persisting questions whether the profile of proposed symptom domains constitute a cohesive constellation and whether adoption of the category has clinical utility beyond the core diagnosis of PTSD. ICD-11 therefore represents a milestone in the field in proposing to include the category of C-PTSD [14], which, apart from the conventional symptoms of PTSD, will include three additional domains of negative evaluation of self or others, affective dysregulation, and interpersonal dysfunction.

There is a small but growing body of evidence supporting the diagnostic criteria of C-PTSD amongst survivors of childhood abuse and sexual assault residing in high-income, largely Anglophone societies [16-20]. In contrast, there is a dearth of research into C-PTSD across cultures, and particularly amongst refugee populations exposed to traumas associated with severe human rights violations. The only relevant study, undertaken amongst conflict-affected populations in Algeria, Ethiopia and Gaza, investigated the category of Disorder of Extreme Stress Not Otherwise Specified (DESNOS), proposed for but not ultimately included in DSM-IV [21]. Significantly, the study failed to identify a unitary construct of DESNOS in these cross-cultural settings. It is timely therefore to examine the structure and antecedents of the reformulated ICD-11 category of C-PTSD amongst a population exposed to persecution and mass violence.

The standard model of PTSD in the refugee and related fields depicts the disorder as being precipitated by exposure to life threatening human rights violations and perpetuated by conditions of ongoing insecurity and related stressors [22,23]. There is growing evidence, however, that the sense of persisting injustice may be instrumental in generating and maintaining the PTSD reaction and related symptoms of distress [24-26]. It may be, therefore, that the sense of injustice distinguishes the unique constructs (negative evaluation of self and others, affect dysregulation, and interpersonal dysfunction) that putatively differentiate C-PTSD from core PTSD.

Our study draws on the Adaptation and Development After Persecution and Trauma (ADAPT) model [27,28] in which it is postulated that amongst refugee populations, five psychosocial domains or pillars that represent the foundations of stable societies are undermined by the often prolonged period of disruption that occurs during the sequence of experiences arising from conflict, displacement and resettlement. These inter-related domains are theorized to comprise safety and security; the integrity of interpersonal bonds and networks; access to effective systems of justice; the ability to pursue roles and maintain identities; and the freedom to pursue activities that confer meaning (spiritual, religious, cultural, political) [28]. We draw selectively on the ADAPT model to examine the contributions of Pillars 1 (safety/security) and 3 (justice) to test for possible distinctions between the outcomes of PTSD and C-PTSD.

The present study was conducted amongst West Papuan refugees displaced to settlements or shanty towns in Port Moresby, Papua New Guinea (PNG). Indonesia occupied West Papua in 1963, annexing the territory in 1969 following a referendum widely regarded as unrepresentative of the wishes of the indigenous people [29]. Widespread resistance to the occupation resulted in a low-grade armed conflict, leading to extensive loss of life and mass internal displacement of the indigenous population [30]. Throughout this prolonged period of conflict, repeated allegations have been made of widespread human rights abuses perpetrated by the occupying Indonesian military, including has perpetrated extensive human rights abuses including extra-judicial arrests, torture, sexual violence and murder [31]. When dissidents are captured, they are held in political prisons under harsh conditions, being subjected to interrogation, torture and gross deprivations. In addition, it is common for community members to be compelled to witness atrocities perpetrated against friends and family members [32]. The prolonged period of repression has had a major impact on the family, the community and the sense of identity of West Papuans. Mass murder, “disappearances” of family members, burning of villages, dispersal of traditional communities, and the influx of emigrants from Indonesia have all acted to threaten the fabric of what has long been a traditional, collectivist society.

Since the 1980s, successive waves of West Papuan refugees, many of whom had been directly involved in the conflict, crossed the border into PNG, with some members of the community settling in shanty towns (“settlements”) in Port Moresby, the capital. As stateless persons, refugees have no right to citizenship, land tenure or ownership. Conditions in the settlements are characterized by extreme poverty, deprivation, and lack of services. West Papuan refugees in Port Moresby have had no contact with services providing interventions for trauma-related mental disorders. Our prior contact with the community reaffirmed that members had no familiarity with western concepts of trauma-related mental disorders such as PTSD.

Our study tested the following hypotheses: (a) that it is possible to identify a unitary PTSD construct with a coherent factorial structure amongst West Papuan refugees; (b) similarly, that a cohesive single structure could be identified for C-PTSD and its constituent subdomains; and (d) that there would be an exclusive relationship between a sense of injustice and the C-PTSD construct and/or its unique subdomains.

## Methods

### Sample

The study sample comprised West Papuan refugees participating in a community survey conducted across six settlements in Port Moresby, Papua New Guinea (PNG). In the absence of census data identifying members of this minority community within the larger population of PNG nationals, a targeted sampling approach was applied. In the first instance, based on all available sources of information (community leaders, government officials, international organizations, local university staff, and the United Nations High Commissioner for Refugees), we identified localities in which West Papuan refugees were concentrated. The six settlements are known as Hohola, Rainbow, Six-Mile, Eight Mile, Nine-Mile, and Tokarara/Waigani, communities characterized by high density, makeshift housing, and few facilities. We estimated that 250 adults (90% of West Papuan refugees living in Port Moresby) resided in these settlements. In the second step, the study team mapped the location of adult refugees within the settlements based on the information already gathered and a comprehensive survey involving door-to-door inquiries, a procedure coordinated by a West Papuan research assistant (MK) from Australia who had long-term contact with the community. Of the 250 eligible respondents, we were unable to contact 20 who had dispersed to other areas of Port Moresby or further afield, yielding a response rate from the identified pool of 92%.

### Measures

#### Exposure to conflict-related traumatic events (TEs)

We assessed exposure to conflict-related traumatic events (TEs) using an inventory of 22 items (rated as experienced or not experienced) complied and adapted to the historical context and experiences of West Papuan refugees in PNG. Development of the item pool was based on an iterative process involving review of the historical and contemporary literature in the refugee field in general, and in relation to West Papua in particular. The list was refined following extensive consultation with the West Papuan refugee community. Exploratory factor analysis was followed by Confirmatory Factor Analyses which yielded five dimensions (χ2 [220] = 241.87, P = 0.149, CFI = 1.00, TLI = 1.00, RMSEA = 0.02): conflict-related trauma (for example, “have you had no shelter or place to sleep because of the conflict?”), traumatic losses (“have you lost your family member during war?”), witnessing murder (“have you witnessed your family member/friend injured or murdered?”), access to emergency medical care (“have you been unable to access medical care when you or a family member was extremely sick?”), and childhood adversities ( “have you been badly beaten as a child?”). The item pool demonstrated sound reliability based on the Kuder-Richardson reliability coefficient (KR20 = 0.94).

### Perceived insecurity and injustice

We assessed refugees’ perceptions of security and injustice related to past persecution using component subscales of a general index of the broader psychosocial effects of conflict and displacement amongst refugee populations [33]. The index comprises five interdependent components or “pillars” derived from the Adaptation and Development After Persecution and Trauma (ADAPT) model. The “pillars” including safety/security; bonds/networks; justice/human rights; roles/identities; and existential meaning dimensions that are postulated to reflect characteristics of the past and present ecosocial environments that are undermined by the effects of mass conflict and displacement. Confirmatory factor analysis supported the designated five dimensions (χ2 [199] = 227.20, P = 0.083, CFI = 0.998, TLI = 0.998, RMSEA = 0.025). Examination of the measurement characteristics of factors across subpopulations of West Papuans (WP- vs. PNG-born) revealed that the ADAPT components showed sound evidence of variance (x2(358) = 632.47, P <0.001, CFI = 0.98, TLI = 0.97, RMSEA = 0.08), providing grounds therefore for focusing on individual subdomains. In the present study we focus specifically on the impacts of perceived ongoing insecurity and injustice related to past human rights violations. Each subscale comprises five core items rated on a four-point Likert scale (0 = none; 1 = little of the time; 2 = some of the time; 3 = most of the time). The items were dichotomized by assigning a summary score of 1 for any item rated 2 (some of the time) or 3 (most of the time).

Focus groups and key informant interviews offered strong support for the constructs and constituent items of safety and justice respectively within the West Papuan context; specifically, participants many expressed a persisting sense of injustice related to past persecution experienced by themselves, their families and others. Psychometric testing based of the subscale items of safety and justice yielded a KR20 of 0.93 and 0.92, respectively, attesting to the reliability of the subscales (items are included in the Additional file 1).

### PTSD symptom measure

We assessed PTSD symptoms using a culturally adapted measure (with each symptom rated as “present” or “absent”). The measure was applied to all respondents following endorsement of a traumatic event according to DSM/ICD definitions. Respondents were asked to respond to symptom all items and a diagnosis was made in the end based on algorithms derived from DSM-IV/5 [10] and ICD-10/11 definitions of PTSD and C-PTSD [14]. Table 1 compares criteria for the relevant diagnostic classification systems for PTSD and C-PTSD. In the present study we focus on symptomatology of PTSD and C-PTSD.

**Table 1:** **Comparison of diagnostic criteria for (complex) PTSD based on DSM-IV, DSM-5, ICD-10, ICD-11**

				DSM-IV	DSM-5	ICD-10	ICD-11	ICD-11
Symptom cluster	Symptoms	Item	Corresponding item	PTSD	PTSD	PTSD	PTSD	C-PTSD
Intrusion	Intrusive thoughts, flashbacks, disturbing dreams	1	Repeated, sudden thoughts about the traumatic experience?	I (at least one symptom, items 1—5)	I (at least one symptom, items 1—5)	I (at least one symptom, items 1—5)		
		2	Repeated disturbing dreams about the experience?	I	I	I	I (at least one symptom, items 2, 3)	I (at least one symptom, items 2, 3)
		3	Suddenly acting as though the experience was happening again?	I	I	I	I	I
	Physical/psychological reactions to reminders of trauma	4	Feeling very upset when reminded of the experience?	I	I	I		
		5	Having strong physical reactions (e.g. dizziness, heart palpitations, chest pain, shortness of breath) when reminded of the event?	I	I	I		
Avoidance	Internal avoidance	6	Avoid thinking about the event?	AN (at least three symptoms, items 6—10, 20, 21)	A (at least one symptom, items 6, 7)	A (at least one symptom, items 6, 7)	A (at least one symptom, items 6, 7)	A (at least one symptom, items 6, 7)
	External avoidance	7	Avoid people, places, talking, activities, things, or situations about the event?	AN	A	A	A	A
Numbing	Diminished interest	8	Losing interest in things you used to enjoy (e.g., walking, reading, socializing, cooking, gardening)	AN	AD			
	Foreshortened future	9	Feeling hopeless about the future	AN				
Hyperarousal	Inability to recall/posttraumatic amnesia	10	Difficulty remembering some important parts of the event?	AN	AD (at least two symptoms, items 8, 10, 17—19, 20, 21)	H (inability to recall or at least two other symptoms, items 10—15)		
	Insomnia	11	Trouble falling or staying asleep?		H	H		
	Irritability	12	Feeling irritable, angry, or aggressive towards people?	H (at least two symptoms, items 12—15)	H (at least two symptoms, items 11—16)	H		AD
	Concentration problems	13	Having difficulty concentrating (e.g., at work/school)?	H	H	H		
	Hypervigilance	14	Being on guard constantly when there was no real reason to be?	H	H	H	H (at least one symptom, items 14, 15)	H (at least one symptom, items 14, 15)
	Exaggerated startle response	15	Feeling suddenly scared for no reason?	H	H	H	H	H
	Self-destructive behaviour	16	Try to do something that you know may cause you or other people harm?		H			
Affect dysregulation	Persistent negative emotions	17	Having strong feelings such as shame, fear, horror, anger, or guilt?		AD			AD (persistent negative emotions)
Negative self-concept		18	Having strong negative beliefs about yourself, others, or the world in general?		AD			NS (at least one symptom, items 18, 19)
		19	Blaming yourself or others constantly for the event or what happened as a result of the event?		AD			NS
Interpersonal problems		20	Feeling cut off or trying to stay away from people?	AN	AD			IP (at least one symptom, items 20, 21)
		21	Having difficulty experiencing positive emotions (e.g. love, happiness) for another person?	AN	AD			IP

DSM-IV, Diagnostic and Statistical Manual for Mental Disorder (4th revision); DSM-5, Diagnostic and Statistical Manual for Mental Disorder (5th revision); CPTSD, complex posttraumatic stress disorder; ICD-10, International Classification of Diseases (10th revision); ICD-11, International Classification of Diseases (11th revision); I = intrusion; A = avoidance; AN = avoidance/numbing; H = hyperarousal; AD = affective dysregulation; NS = negative self-concept; IP = interpersonal problems.

The cultural salience of PTSD as a reaction pattern was examined by a process of qualitative consultation with both community members and local psychiatrists. We commenced the process of development of our measure by assembling a pool of PTSD symptom items drawing on DSM-IV and DSM-5, ICD-10, and the proposed criteria for the forthcoming ICD-11 [14], subjecting the list to further testing of face and construct validity.

Indigenous psychiatrists from PNG reported that they consistently identified symptoms of PTSD amongst persons from Melanesian backgrounds (a culture to which West Papuans belong). Although in focus groups, West Papuans revealed that they had no knowledge of the diagnostic category of PTSD, participants readily recognized the core symptoms of the disorder as reflecting common and problematic experiences within the community. Amongst symptoms recognized were intrusive thoughts, distressing dreams, flashbacks, reactions to triggers of traumatic memories, avoiding thoughts, physiological reactions to cues, startle response, hypervigilance, insomnia, and irritability.

Refinement of the list of PTSD items following the process of consultation yielded 21 items representing PTSD symptoms in the Melanesian context (see Table 2). The derived pool of items was re-tested for their cultural appropriateness in further focus groups involving members of the West Papuan community not involved in the earlier consultation. Participants acknowledged that following exposure to a traumatic event, experiences of cognitive intrusions, nightmares, insomnia, startle responses, and reduced emotional responsiveness were common within the community. Several PTSD symptoms or constellations of symptoms were identified by local terms in the *lingua franca* (Bahasa Indonesian) used by West Papuans. For example, terms commonly referred to were “waspada” (hypervigilance), “menghindari” (avoidance), “kehilangan minat” (loss of interest), “dijaga” (startle response), “sakit hati” (anger and resentment), and “tidak percaya” (loss of trust).

**Table 2:** **Standardized factor loadings and goodness-of-fit statistics for ICD-10/11 derived Confirmatory Factor Analytic (CFA) models of PTSD and C-PTSD in West Papuan refugees**

				PTSD models											
				ICD-10			ICD-11			ICD C-PTSD					
		N	%	F1	F2	F3	F1	F2	F3	F1	F2	F3	F4	F5	F6
	Content														
	Intrusion														
1	Recurring thoughts	94	40.8	1.00											
2	Distressing dreams	54	23.4	0.93			0.94			0.95					
3	Flashbacks	57	24.7	0.91			0.97			0.98					
4	Psychological reactions to cues	73	31.7	0.87											
5	Physiological reactions to cues	57	24.7	0.92											
	Avoidance														
6	Internal avoidance	81	35.2		1.00			1.00			0.98				
7	External avoidance	79	34.3		0.99			0.97			1.00				
	Hyperarousal														
12	Irritability	16	6.9			0.91									
15	Startle response	14	6			0.96			0.98			0.97			
14	Hypervigilance	25	10.8			0.99			0.90			0.97			
13	Concentration problems	17	7.3			0.96									
11	Insomnia	18	7.8			0.98									
	Affective dysregulation														
16	Anger outbursts	16	7										0.92		
17	Negative emotions	19	8.3										0.90		
	Negative self-concept														
18	Distorted beliefs about self or others	11	4.7											0.92	
19	Self-blame	15	6.5											0.97	
	Interpersonal problems														
20	Detachment	24	10.4												1.00
21	Difficulty experiencing positive emotions	18	7.8												0.92
	Goodness-of-fit statistics				First-order	3-factor second-order model	First-order	3-factor second-order model	First-order	6-factor second-order model					
	Second-order factor loadings					F1 = 0.90; F2 = 1.00; F3 = 0.85		F1 = 0.81; F2 = 1.06; F3 = 0.74		F1 = 0.79; F2 = 0.96; F3 = 0.96; F4 = 1.00; F5 = 0.96; F6 = 0.93					
	Chi-square				158.87	61.83		3.43	3.32		38.03	344.39			
	Degree of freedom				35	51		14	6		40	102			
	P				0.07	0.14		0.75	0.76		0.51	<0.001			
	Comparative fit index				0.99	0.99		0.99	0.99		0.99	0.93			
	Tucker Lewis Index				0.99	0.99		0.99	0.99		0.99	0.92			
	Root mean square error of approximation				0.03	0.03		0.00	0.00		0.00	0.00			

The final measure was then applied in interviews in the full survey to all respondents who endorsed experiencing a TE defined according to either DSM or ICD criteria. Once a TE was identified, respondents were asked to respond to all symptom items based on a dichotomous (yes/no) format.

Six months after the baseline study, we followed up a subsample of respondents (n = 101) stratified according to the distribution of symptom scores including those with low (≥ 1), medium (≥ 7), and high (≥ 11) endorsement. We found a high degree of stability in PTSD symptoms over time in that there was no statistical change in the mean symptom score from baseline to follow-up (t1to t2 diff = 0.77, P = 0.149). That finding suggested that symptoms measured at baseline were not simply a reflection of transient distress.

The PTSD item pool demonstrated high internal reliability (KR20 = 0.93 at time 1; KR20 = 0.94 at time 2). In addition, we compared the time 2 PTSD score with that of a standard measure in the field, the Harvard Trauma Questionnaire [34] which is based on DSM-IV. The moderate level of convergence (r = 0.55, p < 0.001) was consistent with expectations in that, unlike the HTQ, our PTSD index included DSM-5 items and had been adapted to the local culture.

### Procedure

Interviews were conducted by West Papuan refugees who received three weeks of intensive training under supervision of a bilingual clinical psychologist focusing on identification of mental health issues amongst trauma survivors, interviewing techniques, role-play, and administration of the assessment protocol. Inter-rater reliability was assessed by the psychologist and a PNG medical practitioner trainee in Psychiatry who independently re-interviewed five study participants who had been assessed by each field worker. There was a high level of interrater agreement in assigning individual diagnoses between field workers and professional personnel (90% overall percentage agreement). Written consent and in some instances, witnessed oral consent were obtained from all participants prior to the interviews. Interviews were conducted in a private location or within the home of the participant, depending on their preference.

Ethical permission for the study was provided by the University of New South Wales Human Research Ethics Committee and the Medical Research Council of PNG Ethics Committee.

### Statistical analysis

Frequency of endorsement and percentages were calculated for individual PTSD symptoms. Confirmatory Factor Analytic (CFA) was conducted based on the DSM-IV and DSM-5 as well as the ICD-10 and proposed ICD-11 symptom constellations for PTSD and C-PTSD. CFA models were estimated using the robust mean- and variance-adjusted Weighted Least Square method (WLSMV), an established statistical procedure recommended for analyzing dichotomous variables [35,36] applied extensively in past studies [37,38]. We tested the DSM-IV three-factor model defined by re-experiencing, avoidance/numbing and hyper-arousal and the DSM-5 four-factor model defined by re-experiencing, avoidance, negative alterations in mood and cognitions, and hyper-arousal. In addition, based on the recent literature examining the DSM-5 symptom constellation [11-13], we tested a series of further factorial solutions, including five-factor (dysphoric-arousal), six-factor (externalizing behaviour), and seven-factor (hybrid anhedonia and externalizing behaviour) models.

We also tested the ICD-10 three-factor model (intrusion, avoidance, and hyperarousal); the ICD-11 three-factor model (based on the smaller number of core symptoms for each equivalent cluster); and the ICD-11 six-factor model of C-PTSD defined by the three PTSD domains of intrusion, avoidance, hyper-arousal, and the additional domains of affective dysregulation, negative self-concept, and interpersonal problems. Where we were able to demonstrate a good fit for a first-order factorial structure, we examined further for a second-order factor as a test of the unitary nature of the overall construct.

#### Multiple-Indicator-Multiple-Causes (MIMIC) analysis

MIMIC modelling is analogous to a multivariate regression in which associations are examined between latent variables (in this case, the factor scores on symptom domains of PTSD) and predictor variables [39]. Specifically, we examined for associations between PTSD/C-PTSD models yielding a good fit in the preceding CFAs and domains of TEs as well as of safety/security and sense of injustice. We commenced the analysis by examining univariate associations between socio-demographic characteristics (sex, age, marital status, education, employment), the five TE domains (conflict-related trauma, witnessing murders, traumatic losses, access to emergency care, childhood adversities), and pillars 1 and 3 of the ADAPT model (safety/security and injustice). Variables (for TEs: conflict-related trauma, witnessing murder, traumatic losses, childhood adversities; for ADAPT: safety/security, justice) producing significant univariate associations (p < 0.05) were included in multivariate models where we tested for the hypothesized interaction effects. The specific interactions we tested were, for TE domains and safety: conflict × safety, witnessing murder × safety, traumatic losses × safety, and childhood trauma × safety; and for traumatic domains and injustice: conflict × injustice, witnessing murder × injustice, traumatic losses × injustice, and childhood trauma × injustice). Our aim was to test for a general relationship involving the sense of insecurity related to the TEs of conflict with PTSD or its subdomains; and whether there was an exclusive relationship between the sense of injustice related to TEs with the unique symptom domains of C-PTSD. The interaction variables were centred for ease of interpreting derived coefficients.

We evaluated model fit using recommended goodness-of-fit and comparative indices, including a non-significant chi-square test, the Confirmatory Factor Index (CFI), Tucker Lewis Index (TLI), and Root Mean Square Error of Approximation (RMSEA). Specifically, a CFI/TLI above 0.95 and a RMSEA below 0.06 indicate a good fit between the model and the data. A moderate fit is indicated by a CFI above 0.90 and a RMSEA below 0.08. In the analysis, we calculated standardized factor loadings and the covariance across factors. In general, a factor coefficient of 0.70 or above is considered to be a reliable indicator of a strongly loaded item; and a cross-factorial correlation of 0.90 or above indicates a high correlation between factors [40,41]. Analyses were performed using STATA version 13 and Mplus version 7 [42,43].

## Results

### Sociodemographic characteristics

The study sample comprised 230 West Papuan adults (men 137, 59.5%; women 93, 40.4%) with a mean age of 37 (sd = 9.80) years. 107 (46.5%) participants originated from West Papua, with the remainder (123, 52.4%) being born in PNG. Participants born in West Papua had lived in PNG for a mean of 27 years (sd = 10.28). Half of participants resided in two settlements: Hohola (65, 28.2%) and Rainbow (47, 20.4%).

### Exposure to traumatic events (TEs)

The majority (n = 129, 56%) reported exposure to at least one type of human right trauma, the order of frequency for TEs being: forced to live in poor conditions during conflict (86, 37.4%); exposure to political upheaval (84, 36.5%); witnessing or hearing about family members and/or strangers being tortured or murdered (78, 33.9%); not being able to access emergency medical care for family members (76, 33%); and traumatic losses involving deaths and disappearances of family members (74, 32.2%).

### ADAPT pillars

Seventy percent (n = 163, 71%) endorsed a sense of insecurity, particularly focusing on safety of family members, and in relation to visiting family or neighbours at night. A high percentage; (n = 162, 70.4%,) reported a sense of injustice and unfairness as a consequence of past persecution and human rights violations.

### Endorsement of PTSD symptoms

Subgroup analyses indicated that PTSD symptoms were associated with age (t(228) = 0.28, P < 0.05). Specifically, older West Papuans who had emigrated from the homeland returned significantly higher PTSD symptom scores (M = 4.18, sd = 0.42) compared to their younger PNG-born counterparts (M = 2.12, sd = 0.36; t(218) = 3.71, P < 0.001).

Table 2 presents endorsement rates of individual PTSD symptoms. Participants endorsed the majority of symptoms, the most widely reported being intrusive thoughts (40.8%), psychological (31.7%) and physiological (24.7%) reactivity, flashbacks (24.7%), distressing dreams (23.4%); internal avoidance (avoiding thoughts) (35.2%) and external (34.3) avoidance (avoiding places, people, and activities). Other reported symptoms included insomnia (7.8%), posttraumatic amnesia (7.8%), affective dysregulation (negative feelings of shame, humiliation, fear, anger) (8.3%), diminished interest (9.1%), and hypervigilance (10.8%).

Overall, 13% (n = 30) of the sample met full criteria for DSM-IV PTSD, and 12% (n = 28) for DSM-5 PTSD. A similar percentage (13%, n = 30) met criteria for ICD-10 PTSD, but only 6% (n = 14) received the diagnosis using ICD-11 PTSD, and 3% (n = 8), C-PTSD.

### Confirmatory factor analysis

The three- and four-factor CFA models based on the respective DSM-IV and DSM-5 definitions of PTSD both yielded a poor fit as indicated by a significant χ^2^ test (DSM-IV: × 2(45) = 189.00, P < 0.01, CFI = 0.99, TLI = 0.99, RMSEA = 0.03; DSM-5: × 2(35) = 158.87, P < 0.001, CFI = 0.99, TLI = 0.99, RMSEA = 0.05). Five, six, and seven-factor CFA models based on the recent literature all showed a poor fit (five-factor: × 2(160) = 202.94, P = 0.012, CFI = 0.99, TLI = 0.99, RMSEA = 0.03; six-factor: × 2(155) = 197.43, P = 0.012; CFI = 0.99, TLI = 0.99, RMSEA = 0.03; seven-factor: × 2(149) = 184.46, P = 0.026; CFI = 0.99, TLI = 0.99, RMSEA = 0.03 [11-13].

In contrast, three-factor CFA models based on both ICD-10 and the proposed ICD-11 definitions of PTSD yielded a good fit (ICD-10: × 2(35) = 158.87, P = 0.07, CFI = 0.99, TLI = 0.99, RMSEA = 0.03; ICD-11: × 2(15) = 3.43, P = 0.75, CFI = 0.99, TLI = 0.99, RMSEA = 0.00). Each of these models was predicted by a higher-order single factor defined by ICD-10 and ICD-11 PTSD respectively (second-order 3-factor ICD-10: × 2(51) = 61.83, P = 0.14, CFI = 0.99, TLI = 0.99, RMSEA = 0.03; ICD-11: × 2(6) = 3.32, P = 0.76, CFI = 0.99, TLI = 0.99, RMSEA = 0.00).

The six-factor model of C-PTSD based on the proposed ICD-11 criteria yielded a good fit: × 2(40) = 38.03, P = 0.51, CFI = 0.99, TLI = 0.99, RMSEA = 0.00). However, the second-order six-factor model yielded a poor fit, suggesting that the individual symptom clusters were heterogeneous and did not form a single coherent structure underlying a unitary construct of C-PTSD (× 2(102) = 344.39, P = <0.001, CFI = 0.93, TLI = 0.92, RMSEA = 0.00). Table 2 reports the goodness of fit statistics for the sequence of CFA models tested.

An examination of the ICD-10 and ICD-11 PTSD constructs indicated that symptoms loaded strongly and predictably on their relevant domains. Standardized factor loadings are presented in Table 2. In relation to the ICD-10 three-factor model, distressing dreams, recurring thoughts, flashbacks, and physiological reactions to cues exhibited strong loadings associated with their designated domains (intrusion, avoidance, hyperarousal) with an overall coefficient > 0.90. Cross-factorial correlations between the three factors were 0.87 (avoidance and hyperarousal), 0.90 (intrusion and avoidance), and 0.87 (intrusion and hyperarousal). The three latent factors loaded strongly on a higher-order structure with each factor demonstrating a high level of loading ranging from 0.85 to 1.00.

The core symptoms of ICD-11 loaded strongly on individual domains (with a coefficient of 0.94) including flashbacks (intrusion), external avoidance (avoidance), and startle response (hyperarousal). CFA indicated low-to-moderate cross-factorial correlations between intrusion and hyperarousal (0.60), intrusion and avoidance (0.86), and avoidance and hyperarousal (0.80). The three latent factors were in turn predicted by a second-order factor.

**Figure 1:**
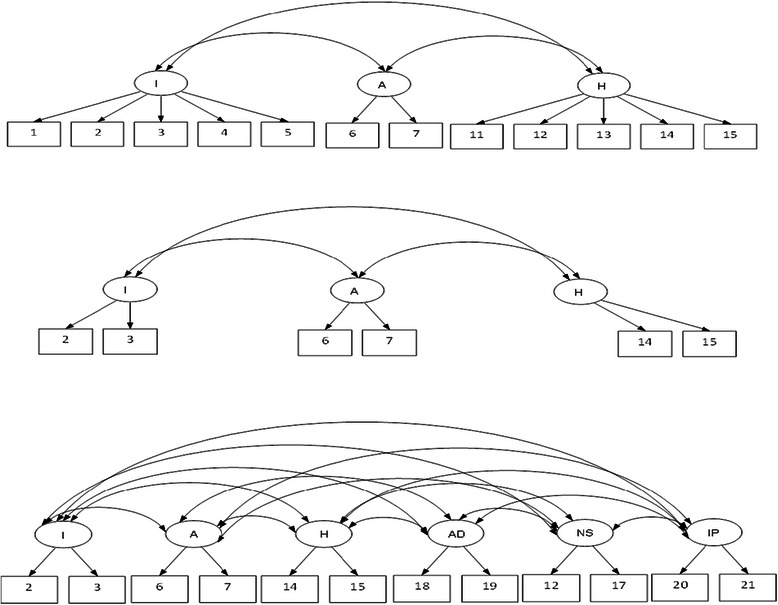
First-order Confirmatory Factor Analytic (CFA) models based on ICD-10 PTSD (top), ICD-11 PTSD (middle) and ICD-11 complex-PTSD.

**Figure 2:**
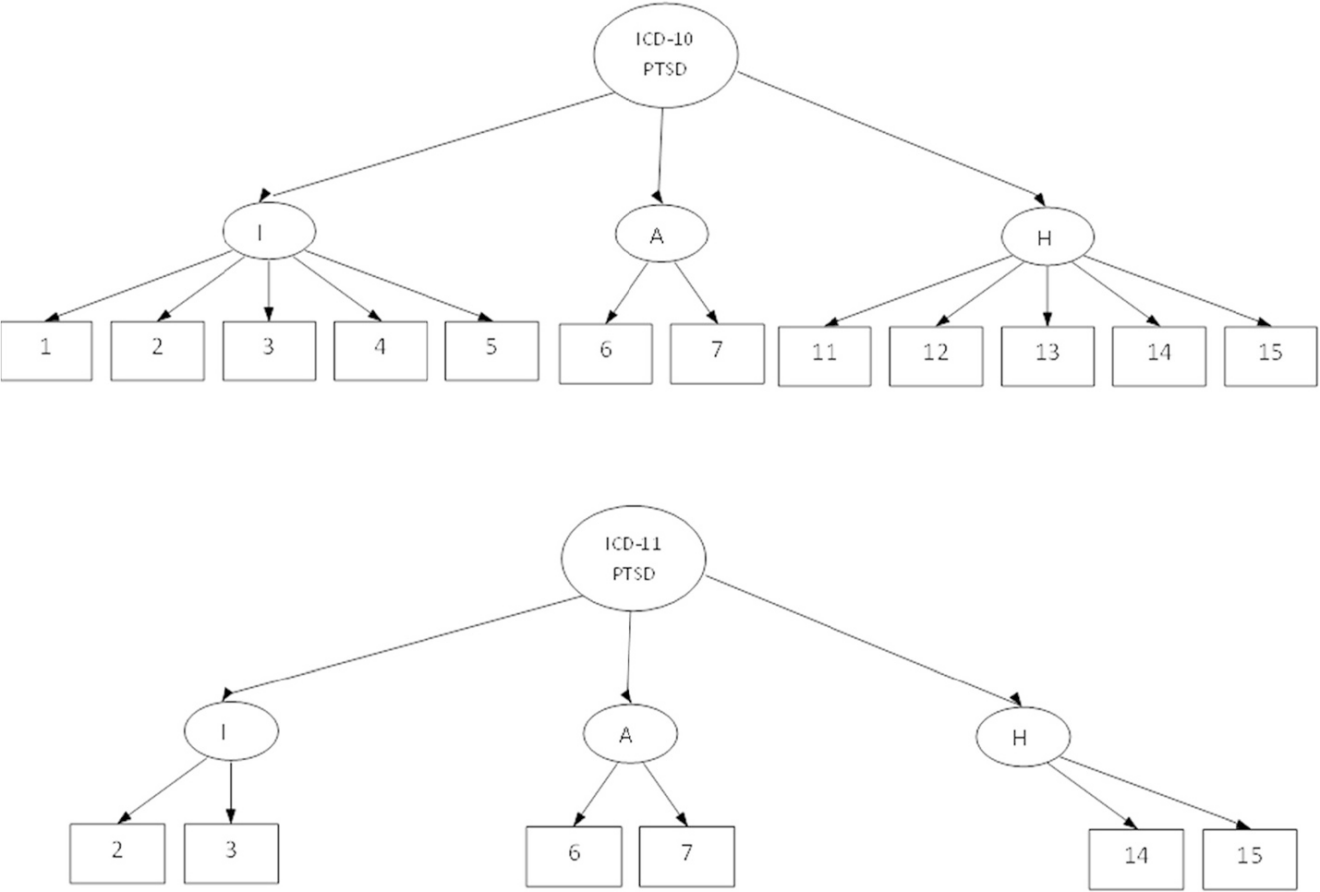
Second-order Confirmatory Factor Analytic (CFA) models based on ICD-10 PTSD (top) and ICD-11 PTSD (bottom).

In relation to the C-PTSD model, the symptoms that demonstrated strong factor loadings included distressing dreams, flashbacks (intrusion); avoidance symptoms; startle response, hypervigilance (hyperarousal); anger outbursts and persistent negative emotional state (affective dysregulation); negative evaluation of self or others, self-blame (negative self-concept); and interpersonal deficits. CFA yielded a high level of correlation between affective dysregulation and hyper-arousal (0.92); and between negative evaluation of self and interpersonal difficulties (0.90). First- and second-order CFA models are represented diagrammatically in Figures 1 and 2.

### Associations with exposure to traumatic events, insecurity, and injustice

Tables 3 and 4 report goodness-of-fit statistics and coefficients for the MIMIC models tested. MIMIC models for ICD-defined PTSD and C-PTSD each yielded a good fit (ICD-10: × 2(87) = 101.14, P = 0.14, CFI = 0.99, TLI = 0.99, RMSEA = 0.02; ICD-11: × 2 (18) = 12.60, P = 0.81, CFI = 0.99, TLI = 0.99, RMSEA = 0.00; C-PTSD: × 2(63) = 57.72, P = 0.66, CFI = 1.00, TLI = 1.00, RMSEA = 0.00).

**Table 3:** **Goodness-of-fit statistics and Multi-Indicators-Multiple-Causes (MIMIC) analyses** ^**a**^ **of predictors of ICD-defined PTSD and C-PTSD symptoms**

	ICD-10 PTSD Symptom clusters								
Predictors	F1: intrusion			F2: avoidance			F3: hyperarousal		
	β	S.E	P	β	S.E	P	β	S.E	P
Gender	−0.37 (−0.14)	0.19	0.06	−0.22 (0–0.09)	0.19	0.25	−0.01 (−0.00)	0.23	0.96
Marital status	0.58 (0.22)	0.19	<0.01	0.19 (0.08)	0.19	0.30	0.13 (0.07)	0.21	0.53
Witnessing murder	0.60 (0.20)	0.24	<0.05	0.64 (0.23)	0.26	<0.05	0.29 (0.13)	0.37	0.43
Witnessing murder x justice	0.06 (0.30)	0.01	<0.001	0.04 (0.24)	0.01	<0.05	0.02 (0.13)	0.02	0.39
Goodness-of-fit statistics									
X2	df	P	CFI	TLI	RMSEA				
101.14	87	0.14	0.99	0.99	0.02				
	**ICD-11 PTSD Symptom clusters**								
**Predictors**	**F1: intrusion**			**F2: avoidance**			**F3: hyperarousal**		
	**β**	**S.E**	**P**	**β**	**S.E**	**P**	**Β**	**S.E**	**P**
Gender	0.11 (−0.05)	0.19	0.55	−0.23 (−0.05)	0.20	0.25	0.44 (−0.05)	0.49	0.36
Marital status	0.41 (0.18)	0.19	<0.05	0.20 (0.18)	0.19	0.30	0.35 (0.17)	1.34	0.18
Witnessing murder	0.76 (0.28)	0.24	<0.01	0.65 (0.28)	0.27	<0.05	0.44 (0.19)	0.49	0.36
Witnessing murder x justice	0.05 (0.28)	0.01	<0.01	0.04 (0.28)	0.01	<0.05	−0.02 (0.13)	0.03	0.50
Goodness-of-fit statistics									
X2	df	P	CFI	TLI	RMSEA				
12.60	18	0.81	0.99	0.99	0.00				

SE = standard errors; β = standardized coefficients are presented in parentheses; ^a^ MIMIC analyses adjusted for significant covariates identified in univariate analyses: sex marital status; for TEs, conflict-related trauma, witnessing murders, traumatic losses, childhood related adversities; for ADAPT, safety/security, bonds/networks, justice, existential meaning.

**Table 4:** **Goodness-of-fit statistics and Multi-Indicators-Multiple-Causes (MIMIC) analyses** ^**a**^ **of predictors of ICD-defined PTSD and C-PTSD symptoms**

	ICD-11 C-PTSD																	
	Symptom clusters																	
Predictors	F1: intrusion			F2: avoidance			F3: hyperarousal			F4: affective dysregulation			F5: negative self-concept			F6: interpersonal problems		
	β	S.E	P	β	S.E	P	β	S.E	P	β	S.E	P	β	S.E	P	β	S.E	P
Gender	−0.12	0.20	−0.55	−0.23	0.20	0.25	−0.02	0.26	0.94	0.06	0.24	0.25	−0.27	0.30	0.36	−0.17	0.25	0.49
	(−0.05)			(−0.09)			(−0.01)			(0.03)			(0.08)			(−0.08)		
Marital status	0.42	0.19	<0.05	0.20	0.19	0.30	0.09	0.25	0.72	0.15	0.25	0.52	0.34	0.26	0.18	0.24	0.23	0.31
	(0.18)			(0.08)			(0.04)			(0.08)			(0.08)			(0.11)		
Witnessing murder	0.77	0.24	<0.01	0.65	0.27	<0.05	0.36	0.44	0.41	0.10	0.39	0.78	0.43	0.47	0.36	0.38	0.47	0.41
	(0.28)			(0.23)			(0.14)			(0.04)			(0.04)			(0.15)		
Witnessing murder x justice	0.05	0.01	<0.05	0.04	0.01	<0.05	0.02	0.02	0.40	0.02	0.02	0.42	−0.02	0.03	0.51	−0.00	0.02	0.77
	(0.28)			(0.24)			(0.13)			(0.14)			(0.14)			(−0.05)		
Goodness-of-fit statistics																		
X2	df	P	CFI	TLI	RMSEA													
57.72	63	0.66	1.00	1.00	0.00													

^a^MIMIC analyses adjusted for significant covariates identified in univariate analyses: sex marital status; for TEs, conflict-related trauma, witnessing murders, traumatic losses, childhood related adversities; for ADAPT, safety/security, bonds/networks, justice, existential meaning.

**Figure 3:**
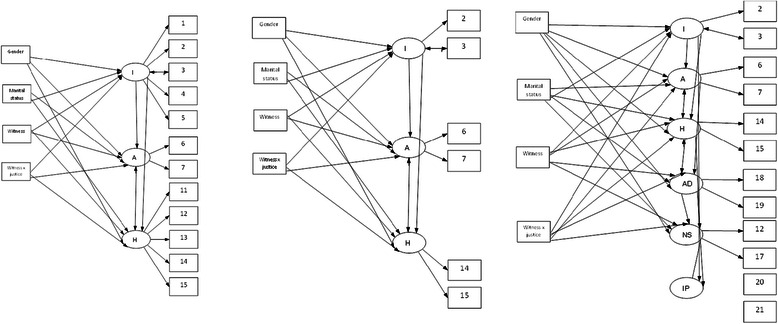
Multi-Indicators-Multiple-Causes (MIMIC) models examining predictors of symptom domains of ICD-10 PTSD (left), ICD-11 PTSD (middle), and ICD-11 complex-PTSD (right).

Witnessing murder and the interaction of witnessing murder x injustice were strongly associated with the intrusion and avoidance symptom domains of PTSD based on both ICD-10 and ICD-11 definitions, and with the same domains for ICD-11 C-PTSD, after adjusting for other covariates. Marital status, specifically being single, was a significant predictor of intrusion symptoms of PTSD and C-PTSD. MIMIC models are represented diagrammatically in Figure 3.

## Discussion

Our findings, based on a transcultural population, support a unitary PTSD construct consistent with ICD-10 and ICD-11 criteria, comprising the three core symptom domains of intrusions, avoidance, and hyper-arousal. In contrast, we could not confirm a unitary construct underlying the DSM-IV or DSM-5 definitions of PTSD. In addition, the five, six, and seven-factor models based on DSM-5 symptoms reported for populations originating in the US all yielded a poor fit [11-13]. Although coherent factors emerged for the individual domains of the proposed ICD-11 category of C-PTSD, our analysis failed to identify a unitary higher order factor underlying the constellation as a whole. Our findings therefore add to long-standing concerns that the domains proposed in formulations of complex PTSD do not cohere to form a unitary construct, one of the vital prerequisites for a constellation to warrant recognition as a diagnostic entity. Associations emerged for witnessing murder on its own and interacting with injustice with the intrusion and avoidance but not with the hyperarousal domains of both ICD PTSD and C-PTSD. Importantly, however, there were no associations of traumatic domains or injustice with the proposed three unique symptom clusters of C-PTSD.

Prior to discussing our findings, we consider the strengths and limitations of the study. Given the absence of census data identifying West Papuan refugees and the dispersal of this minority within a larger pool of PNG nationals, we used all available information sources to estimate the size and location of the target population in the settlements in Port Moresby. The response rate from the pool identified was high; only a small number of West Papuans known to the community were not traceable. We applied a culturally adapted checklist to assess PTSD symptoms according to the DSM and ICD criteria. Limiting symptoms to DSM and ICD classification systems inevitably precluded a detailed analysis of culture-specific idioms of distress that may overlap with symptoms of PTSD.

We note that only a small number of persons met full diagnostic criteria for C-PTSD. Our analysis was based, however, on dimensional data to identify the relevant symptom domains across the population as a whole and there was sufficiently high endorsement of constituent items to detect coherent individual factors as proposed in the formulation of C-PTSD. The negative finding, however, was that we were unable to identify a single higher order factor reflecting a cohesive C-PTSD construct.

The study is cross-sectional, cautioning against drawing causal inferences concerning the association between past TEs, the sense of insecurity and injustice, and current symptom domains. Given the specific experiences of the community in the settlements in Port Moresby, we cannot generalize the findings to West Papuan populations residing in the home country or further afield. Our index of PTSD was validated specifically amongst the West Papuan community in Port Moresby, a culturally distinct group with no exposure to western concepts of trauma. These factors may account in part for the difference between our findings and those of factorial studies undertaken amongst populations exposed to other forms of trauma residing in high-income countries. Further studies are needed therefore to examine the influence of type of trauma, culture and context in identifying the factorial structure of PTSD.

Notwithstanding these caveats, our study identified a unitary construct of PTSD consistent with both ICD-10 and ICD-11 definitions. In contrast, we found no evidence for a unitary construct underlying either the DSM-IV or DSM-5 definitions of PTSD or ICD-11 C-PTSD. The distinctions we found are of particular interest given that there is a paucity of evidence supporting the universal applicability of the DSM-IV or DSM-5 definitions of PTSD across transcultural populations, even though the relevant criteria have been widely applied in these settings. The DSM-IV definition of PTSD includes avoidance and numbing symptoms (e.g., social detachment, foreshortened future, posttraumatic amnesia), and these features also appear in the definition of DSM-5, which, however, has been expanded to include the additional domain of affective dysregulation (including strong negative beliefs about oneself; feelings of guilt, shame, anger; self-blame; and self-destructive behaviour). In contrast, ICD-10 and the proposed ICD-11 definitions of PTSD are consistent in being limited to the three clusters thought to be core to the learned fear response [44], that is, re-experiencing (or intrusions), avoidance, and hyper-arousal, the most recent revision containing a reduced number of symptoms for each domain. Consistent with a recent study amongst traumatic injury survivors in a western, developed country [45], our study identified a higher number of persons meeting the ICD-10 definition (13%) compared to ICD-11 criteria (6%) of PTSD, adding face validity to our findings.

Our CFA analysis identified individual symptom domains proposed for C-PTSD, suggesting that each factor does form a coherent reaction pattern in its own right. Importantly, however, the absence of a higher order factor argues against C-PTSD constituting a coherent, unitary construct, at least amongst this cross-cultural population. Our findings therefore are consistent with the negative results of an earlier study undertaken amongst three post-conflict, culturally-diverse populations, examining the putative category of Disorders of Extreme Stress (DESNOS) [21], the forerunner of C-PTSD identified but not included in DSM-IV. It is noteworthy too that studies supporting the structure of C-PTSD have been limited to survivors of childhood abuse and sexual assault in western countries [16-18]. Our data casts doubt therefore on the appropriateness of extending the C-PTSD category to trauma-affected refugees from diverse cultures, although individual components, such as explosive forms of anger, may be highly relevant to these populations.

It is noteworthy that our study failed to demonstrate an association between childhood adversities and domains of PTSD/C-PTSD. These findings may suggest either that childhood adversity does not show the same pattern of relationship with mental disorder in the developing world or, alternatively, that, in traditional societies, respect for parents results in under-reporting of adverse childhood experiences or a tendency not to link these events to adverse adult mental health outcomes. We note, however, that our measure of early adversity was limited in scope, cautioning against drawing any inference that childhood adversities are not associated with PTSD/C-PTSD in this transcultural setting.

The key positive finding of our analysis was the robust evidence it yielded supporting the unitary ICD-10 and ICD-11 structure comprising the three symptom domains of intrusions, avoidance and hyper-arousal in this transcultural population that has had no prior exposure to formal mental health services focusing on traumatic stress. As such, our findings offer support for the ICD definition (represented by both ICD-10 and ICD-11) as representing the core universal PTSD reaction pattern [46]. Cumulative data from other culturally distinct populations offers supportive evidence for this possibility [8].

Our demonstration that witnessing murder of families on its own and interacting with feelings of injustice were the trauma-related experiences associated with intrusions and avoidance adds further evidence in support of these two domains being core to the PTSD constellation. In general, political persecution has been shown to be a potent factor in shaping the PTSD response [47]. In the West Papuan context, repeated allegations have been made that forcing family and community members to witness the abuse and murder of others is key to the overall campaign of repression perpetrated against the community as a whole. In a traditional collectivist society, such experiences are likely to engender particularly strong and persisting feelings of injustice which we found to interact with the core experience of witnessing murder in engendering the PTSD reaction. A similar pattern has been described in comparable settings of conflict and mass persecution in other countries [24,26].

The finding that being single was associated with PTSD remains to be explained, suggesting the need for further qualitative inquiry to cast light on this observation. It is possible that marital status represents a proxy index of age in that younger single adults were less exposed to traumatic events in West Papua and less likely to experience PTSD symptoms. This is substantiated by our further subgroup analyses suggesting that there was a positive association between age and PTSD symptoms, in that older West Papuans who are refugees returned a higher mean PTSD symptom score compared to their younger counterparts born in PNG.

In summary, our findings offer support for the construct validity of ICD-10 and ICD-11 definitions of PTSD in a transcultural population that has had limited contact with western psychiatric concepts or services. Our findings therefore pose a challenge to the assertion that the PTSD construct is culture-bound to western societies [5]. To the contrary, our analysis suggests that exposure to extreme forms of human rights violations, particularly witnessing murder, together with associated feelings of injustice, can have an enduring impact on symptoms of PTSD across cultures. Addressing ongoing feelings of injustice therefore may prove critical to interventions aimed at reducing symptoms of PTSD amongst refugee populations such as the one under study from West Papua. From a clinical perspective, there may be benefits in expanding the scope of culturally-adapted forms of trauma-focused treatments for refugees to include interventions that address feelings of injustice, for example, those associated with traumatic losses, an experience whose impact may be magnified in collectivist societies such as the indigenous peoples from West Papua.

## Conclusions

Our study provides added support for the transcultural relevance of three core symptom domains of intrusion, avoidance, and hyper-arousal underlying the construct of PTSD, by demonstrating their salience amongst a West Papuan refugee population with no past exposure to formal western mental health services. Two of these domains (intrusions and avoidance) were specifically associated with witnessing murder of families and others on its own and as those experiences interact with a persisting sense of injustice. Importantly, although the individual domains of C-PTSD could be identified, we could not find evidence of a unitary construct underpinning this category in this transcultural setting.

## Additional file

Additional file 1:
**ADAPT subscales.**
