# Supplementary material for: The structure of post-traumatic stress disorder and complex post-traumatic stress disorder amongst West Papuan refugees
Source: BMC Psychiatry. 2015 May 7;15:111. doi: 10.1186/s12888-015-0480-3 (PMC4459680; doi:10.1186/s12888-015-0480-3)
Supplement: Additional file 1: — ADAPT subscales. [file 12888_2015_480_MOESM1_ESM.docx]

**Additional files**

**Additional file 1 – ADAPT subscales**

**ADAPT Pillar 1: safety and insecurity**

1. In the 12 months, how often have you had serious concerns about whether family will survive? **[1 none/ 2 little of the time/ 3 some of the time/ 4 most of the time]**
2. In the 12 months, how often have you felt unsafe about visiting family or neighbours? **[1 none/ 2 little of the time/ 3 some of the time/ 4 most of the time]**
3. In the 12 months, how often have you had serious concerns about my family might die of hunger? **[1 none/ 2 little of the time/ 3 some of the time/ 4 most of the time]**
4. In the 12 months, how often have you had serious worries that the future will be insecure? **[1 none/ 2 little of the time/ 3 some of the time/ 4 most of the time]**
5. In the 12 months, how often have you felt very nervous about family members getting sick because unable to afford medical care? **[1 none/ 2 little of the time/ 3 some of the time/ 4 most of the time]**

**ADAPT Pillar 3: Access to justice for past human rights violations**

1. In the 12 months, how often have you had strong feelings of unfairness about the way me and family had been treated in the past? **[1 none/ 2 little of the time/ 3 some of the time/ 4 most of the time]**
2. In the 12 months, how often have you thought excessively about the unjust things that happened to me and family in the past? **[1 none/ 2 little of the time/ 3 some of the time/ 4 most of the time]**
3. In the 12 months, how often have you lost trust in people because of the unjust things that happened to me and my family in the past? **[1 none/ 2 little of the time/ 3 some of the time/ 4 most of the time]**
4. In the 12 months, how often have you become suspicious of authorities because of the unjust things that happened to me and my family in the past? **[1 none/ 2 little of the time/ 3 some of the time/ 4 most of the time]**
5. In the 12 months, how often have you had difficulties accepting the unjust things that happened to me and my family? **[1 none/ 2 little of the time/ 3 some of the time/ 4 most of the time]**
